# New molecular settings to support in vivo anti-malarial assays

**DOI:** 10.1186/s12936-016-1205-x

**Published:** 2016-03-08

**Authors:** Noemí Bahamontes-Rosa, Ane Rodriguez Alejandre, Vanesa Gomez, Sara Viera, María G. Gomez-Lorenzo, Laura María Sanz-Alonso, Alfonso Mendoza-Losana

**Affiliations:** Diseases of the Developing World, GlaxoSmithKline, Tres Cantos, 28760 Madrid, Spain

**Keywords:** Malaria, *Plasmodium falciparum*, *Plasmodium yoelii*, *Plasmodiumberghei*, Quantitative real-time PCR, qPCR, FTA

## Abstract

**Background:**

Quantitative real-time PCR (qPCR) is now commonly used as a method to confirm diagnosis of malaria and to differentiate recrudescence from re-infection, especially in clinical trials and in reference laboratories where precise quantification is critical. Although anti-malarial drug discovery is based on in vivo murine efficacy models, use of molecular analysis has been limited. The aim of this study was to develop qPCR as a valid methodology to support pre-clinical anti-malarial models by using filter papers to maintain material for qPCR and to compare this with traditional methods.

**Methods:**

FTA technology (Whatman) is a rapid and safe method for extracting nucleic acids from blood. Peripheral blood samples from mice infected with *Plasmodium berghei*, *P. yoelii*, or *P. falciparum* were kept as frozen samples or as spots on FTA cards. The extracted genetic material from both types of samples was assessed for quantification by qPCR using sets of specific primers specifically designed for *Plasmodium* 18S rRNA, LDH, and CytB genes.

**Results:**

The optimal conditions for nucleic acid extraction from FTA cards and qPCR amplification were set up, and were confirmed to be suitable for parasite quantification using DNA as template after storage at room temperature for as long as 26 months in the case of *P. berghei* samples and 52 months for *P. falciparum* and *P. yoelii*. The quality of DNA extracted from the FTA cards for gene sequencing and microsatellite amplification was also assessed.

**Conclusions:**

This is the first study to report the suitability of FTA cards and qPCR assay to quantify parasite load in samples from in vivo efficacy models to support the drug discovery process.

## Background

In anti-malarial clinical trials discerning between mixed infections, resistance, re-infection and recrudescence, is crucial to establish the efficacy of new anti-malarials. The World Health Organization highly recommends the use of molecular analysis techniques, like PCR, to assist in distinguishing recrudescence from re-infection [1], and several methods have been implemented so far for this purpose [2]. In clinical trials, a cheap and long-term storage method is used for biological samples. The FTA technology (Whatman) consists of index-sized cards developed for collection and storage of organic samples from field isolates. FTA cards are routinely used to support clinical trials because they are safe and easy to transport at room temperature between laboratories, and the genetic material of the parasite remains unchanged in the spot sample for long-term storage [3, 4]. However, no research has been performed so far to translate the use of this technique to mouse malaria models. In drug discovery, the most common murine *Plasmodium* species used are *Plasmodium berghei* and *P. yoelii* and, a closer subrogate for human parasite infection, the humanized mouse model with *P. falciparum* [5–7].

Among molecular detection techniques, quantitative real-time PCR (qPCR) has demonstrated a high sensitivity for detection of low parasite burdens, and is now commonly used as a method to confirm diagnosis of malaria, especially in clinical trials and in reference laboratories where precise quantification is critical [8–10]. This is the first study to report parasite identification and quantification using FTA blood spots to support pre-clinical animal models. It was designed to identify the adequate molecular settings that may be encountered across laboratories to support anti-malarial in vivo assays.

## Methods

### Parasites and animal models

The *Plasmodium* species tested as tool organisms for drug screening were the human pathogen *P. falciparum* and the mouse malaria parasite species *P. yoelii* and *P. berghei*.

*Plasmodium falciparum* Pf3D7^0087/N9^ was maintained continuously in vivo in non-myelodepleted NOD-scid IL2R gamma-null mice obtained from Charles River Laboratories, engrafted with human erythrocytes [5]. *Plasmodium yoelii* and *P. berghei* were maintained by in vivo passage on CD1 mice purchased from Harlan Laboratories. Parasitaemia from mouse malaria models was routinely monitored on peripheral blood by flow cytometry [11]. Protocols were approved by the DDW Ethical Committee on Animal Research, performed at the DDW Laboratory Animal Science facilities accredited by AAALAC, and conducted according to European Union regulations and GlaxoSmithKline policy on the care and use of animals.

### Biological material

*Plasmodium falciparum*-infected blood samples were taken from the tail vein of the NOD-scid IL2R gamma-null engrafted mice, and blood samples infected with *P. berghei* or *P. yoelii* were taken from CD1 mice. Parasitaemia was measured by flow cytometry. To establish the limit of detection (LOD), *P. falciparum* blood samples were serially diluted tenfold using fresh AB+ human erythrocyte concentrates generously donated by the Spanish Red Cross blood bank, and *P. berghei* and *P. yoelii* blood samples were diluted with uninfected blood from a donor CD1 mouse. Non-infected human RBC samples were included as negative controls for *P. falciparum* samples, and mouse blood samples as negative controls for *P. berghei* and *P. yoelii*. Fifty µl from all samples were immediately stored at −80 °C, and 20 µl spotted onto an FTA card (GE Healthcare), dried for 3 h at room temperature (RT), and stored at RT from 1 day up to 26 months for *P. berghei* samples and for 52 months for *P. yoelii* and *P. falciparum* for further DNA extraction. The lowest concentration of DNA that tested positive in all replicates was considered the LOD.

### DNA extraction

To extract DNA, the DNeasy Blood and Tissue Kit (Qiagen) was used. For frozen samples, the manufacturer recommendations were followed. For DNA isolation from the FTA cards, the complete disk was punched out from each filter paper and incubated with the lysis buffer provided by the manufacturer at 56 °C for 1 h, followed by incubation for 10 min at 70 °C in shaking conditions. Lysed infected red blood cells (iRBC) were then placed onto the purification column provided, and purification continued according to the standard protocol suggested by the manufacturer.

### Genes used

The *Plasmodium* 18S ribosomal RNA (18S rRNA), lactate dehydrogenase (LDH), and cytochrome B (CytB) genes chosen for the study encode highly conserved proteins and are considered as housekeeping genes in detection and quantification studies [12–14]. Gene sequences were retrieved from PlasmoDB and used as templates to design qPCR primers using Primer Express Software v.1.5 (Applied Biosystems). Primer sequences are described in Table 1. Primers already reported in the literature for *P. falciparum* LDH, *P. yoelii* 18S rRNA, and *P. berghei* LDH and CytB primers were also used in this study [12–15].

**Table 1:** Genes and primer sequence

*P. falciparum*	
18S rRNA	Pf_18S rRNA-F: AATAACAATGCAAGGCCAATTT
	Pf_18S rRNA-R: CTGCAACAATTTTAATATACGC
LDH	Pf_LDH-F: AAACCCAGTAGATGTTATGGTAC
	Pf_LDH-R: CATTTTATTTCCATGAGCACCTAC
CytB	Pf_CytB-F: ACTTGTTATCCTCTATTCCAGTAGCAGTAA
	Pf_CytB-R: AATCGTTTTATTGTAGGATCACTCACA
*P. yoelii*	
18S rRNA	Py_18S rRNA-F: GCAACTCACTTGGCTAGATTCTT
	Py_18S rRNA-R: CGGCTTTAACTGTTTGCTTTG
LDH	Py_LDH-F: GCTCGACTTTATTAGAAGGCCAAT
	Py_LDH-R: TCGATTACTTGTTCTACACCATTACCA
CytB	Py_CytB-F: TTTACATTTACATGGTAGCACTAATCCTTT
	Py_CytB-R: CCTTTTACATCAAGACTTAATAGATTTGGA
*P. berghei*	
18S rRNA	Pb_18S rRNA-F: AAGCATTAAATAAAGCGAATACATCCTTAC
	Pb_18S rRNA-R: GGAGATTGGTTTTGACGTTTATGTG
LDH [15]	Pb_LDH-F: GGAAGGAGGTTCACAAGCAGGT
	Pb_LDH-R: TATGCTCTCAGCCAAGTCTGCC
CytB [14]	Pb_CytB-F: TGGGGACAAATGAGTTACTGG
	Pb_CytB-R: CAGTGTATCCTCCACATAACCAA

*LDH* lactate deshydrogenase, *CytB* cytochrome b

### Quantitative real-time PCR

Quantitative real-time PCR was performed using an ABI PRISM 7500 fast sequence detection system (Applied Biosystems). Reagents used included Sybr green JumpStart Taq ReadyMix (Sigma); 250 nM forward primer; 250 nM reverse primer; and distilled sterile water up to a total reaction volume of 13 + 2 μl of the genetic material. Reaction mixtures were prepared at RT in a 96-well optical reaction plate (Applied Biosystems) covered with optical adhesive covers (Applied Biosystems). The thermal cycling conditions were 50 °C for 2 min, 95 °C for 10 min and 40 cycles of 95 °C for 15 s and 60 °C for 1 min. Amplification of each gene was done in duplicate, and the average value was taken as the result. Ct values are inversely proportional to the amount of template in the sample and were used to quantify the relative amount of target PCR product present in each reaction. Only duplicate Ct values with standard deviation within ±1.5 Ct were accepted. All qPCR assays were run including the Non-Template Control (NTC), and melting curve analysis was always performed at the end of each assay as a specificity control.

### Parasite genotyping

Genotyping of *P. falciparum* Pf3D7^0087/N9^ strain was done by amplification of the microsatellite regions from DNA extracted from the FTA cards. The technique, based on the multiple and species-specific repetition of microsatellite sequences, was described by Su et al. in 1998 using specific primers labeled with FAM (FFF: TAACGTTACATTATGTTTTA-FAM and FFR: ATATGGTATTGCGCTTTTA) [16].

### Amplification and sequencing of *P. falciparum* CytB gene

The protocol used was an adaptation from the one reported by Korsinczky et al. [17]. The CytB gene was amplified from DNA extracted from the FTA cards using primers F CYTB and R CYTB that anneal outside the ORF (primer sequences in Table 2). PCR was performed using 2 µl of DNA as template. PCR products were purified and then sequenced using the ABI Prism Big-Dye Terminator kit (Applied Biosystems). Nine internal primers (forward and reverse) were then used to sequence the amplified products (Table 2).

**Table 2:** Primer for amplification and sequencing of the *P. falciparum* CytB gene

Primer for amplification	
F CYTB	CCTGATTATCCAGACGCTTTAAATGG
R CYTB	CATGTCTTGCTAACGGCTTGTACGG
Primer for sequencing	
BC1	AACGGTGTATTTTTAGCAAGTGG
BC2	GAATAAAAAATATTATTCCTAAAAGG
BC3	CAAATGAGTTATTGGGGTGCAAC
BC4	TCATAAATAAAATCAATCCAGATATCC
BC5	TATCCAAATCTATTAAGTCTTGATG
BC6	AATGCTGTATCATACCCTAAAGG
BC7	GGTGCTAGAGATTATTCTGTTCC
BC8	TGTTCTGCTAATAAGAATAATAATTG
BC9	AACACATTATGATTACAGCTCC

## Results

### Suitability of FTA for in vivo anti-malarial studies

**Fig. 1:**
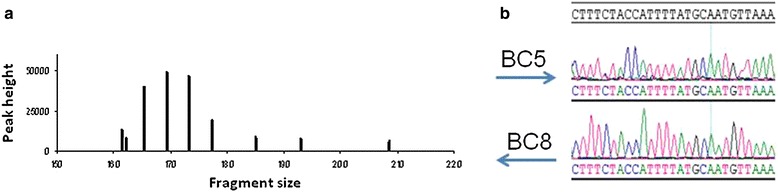
Assessment of the quality of the nucleic acids extracted from FTA cards. **a** Genotyping of *P. falciparum* Pf3D7^0087/N9^ was performed using the procedure based on multiple and species-specific repetitions of microsatellite sequences described by Su et al. in 1998. **b** Electropherogram showing nucleic acid alignment using SeqMan software and sequenced with primers BC5 and BC8. The CytB 3D7 *P. falciparum* genome sequence M76611 (PlasmoDB) was used as reference

The value of FTA cards for collecting and storing samples from in vivo animal anti-malarial studies was assessed. DNA from the filter papers were recoverd using a silica-membrane-based column method. Optimal DNA yield and quality for direct qPCR analysis was obtained following the protocol described here. In order to further assess the quality of the nucleic acids extracted from FTA cards, the species-specific repetition of microsatellite was successfully amplified, giving the same pattern as previously reported for the *P. falciparum* strain used in the mouse model (Fig. 1a) [18]. Using the same extracted genetic material, the Pf CytB gene was sequenced, finding no mutations as compared to the CytB 3D7 *P. falciparum* genome sequence M76611 (PlasmoDB). The peaks from the electropherograms were single and clear for each nucleotide without uncertainty, with a low background line (Fig. 1b). These results showed that the extracted genetic material had enough quality to support identification of specific resistance determinants in long DNA stretches or to assess identification of mixed infections.

### qPCR setup conditions

The optimal qPCR amplification conditions were set up using DNA obtained from tenfold serial fresh blood samples diluted with human erythrocyte concentrates for *P. falciparum* and with uninfected blood from a donor mouse for *P. berghei* and *P. yoelii.* Each assay was performed in duplicate, and efficiency of amplification with the primer sets was calculated. A linear regression plot was generated and the slope, Y-intercept, and r^2^ value were calculated. Data shown in Table 3 confirm that all primers showed suitable conditions for parasite quantification and efficiencies ranging from 94.88 to 50.46 %, with r^2^ ranging from 0.99 to 0.92.

**Table 3:** qPCR assay performance

Gene	Slope	Y-Inter	R^2^	% Eff
*P. falciparum*				
LDH	−3.77	27.03	0.99	84.33
CytB	−3.80	16.49	0.99	83.38
18S rRNA	−3.56	5.69	0.99	90.92
*P. yoelii*				
LDH	−3.45	21.62	0.95	94.88
CytB	−3.70	13.97	1.00	86.28
18S rRNA	−3.73	16.65	0.99	85.49
*P. berghe*				
LDH	−4.27	23.30	0.96	71.43
CytB	−5.64	22.53	0.92	50.46
18S rRNA	−4.55	25.42	1.00	65.93

The efficiency of each qPCR was calculated with the formula: Efficiency = −1 + 10(−1/slope) (http://www.thermoscientificbio.com/webtools/qpcrefficiency/), which uses the slope produced by a qPCR standard curve. qPCR efficiency was validated by comparing the slope of the standard curve to the theoretical optimum of −3.32 which reflects 100 %

### Parasite LOD calculation by qPCR

For parasite detection, LDH, 18S rRNA, and CytB genes were evaluated using the corresponding strain-specific primers shown in Table 1. The LOD of the qPCR assay was determined from DNA extracted from frozen blood samples and embedded in FTA cards. All assays were performed in duplicate, and the LOD was established as the highest Ct value at which the lowest parasite concentration was detected and expressed as log10 parasites/µl. Only Ct values below 40 and duplicate Ct values with standard deviation within ±1.5 Ct were considered. The mean LOD values are given in Table 4 and varied depending on the source and genes used. Assay fluctuation included variation in both nucleic acid extraction and amplification. DNA from FTA cards was successfully obtained and even a parasite burden lower than one parasite/µl (negative log10 parasites/µl) was detected with *P. falciparum* CytB gene. Similar detections were obtained in *P. berghei* with the CytB and 18S rRNA genes and in *P. yoelii* with the 18S rRNA gene. When LOD values were compared between frozen samples and FTA cards, a lower detection limit was seen for the CytB gene in samples stored in filter paper. For frozen samples, higher sensitivity was seen for the LDH and 18S rRNA genes.

**Table 4:** Limit of detection (log10 parasites/µl)

Gene	DNA frozen	DNA FTA
*P. falciparum*		
LDH	2.2 (1.1 to 3.2)	4.9 (4.5 to 5.2)
CytB	1.9 (1.1 to 2.6)	0.4 (−0.8 to 1.5)
18S rRNA	1.9 (0.1 to 3.6)	3.9 (2.5 to 5.2)
*P. yoelii*		
LDH	0.8 (0.8 to 0.8)	2.2 (0.2 to 4.2)
CytB	2.8 (0.8 to 4.8)	1.5 (0.9 to 2.2)
18S rRNA	0.3 (−0.2 to 0.8)	1.2 (0.2 to 2.2)
*P. berghei*		
LDH	0.9 (0.9 to 0.9)	1.8 (1.3 to 2.3)
CytB	0.9 (−0.1 to 1.9)	0.8 (0.3 to 1.3)
18S rRNA	0.4 (−1.1 to 1.9)	2.8 (2.3 to 3.3)

Detection limit of log10 parasites/µl expressed as the median of the experiments (min–max)

### FTA long-term storage

**Fig. 2:**
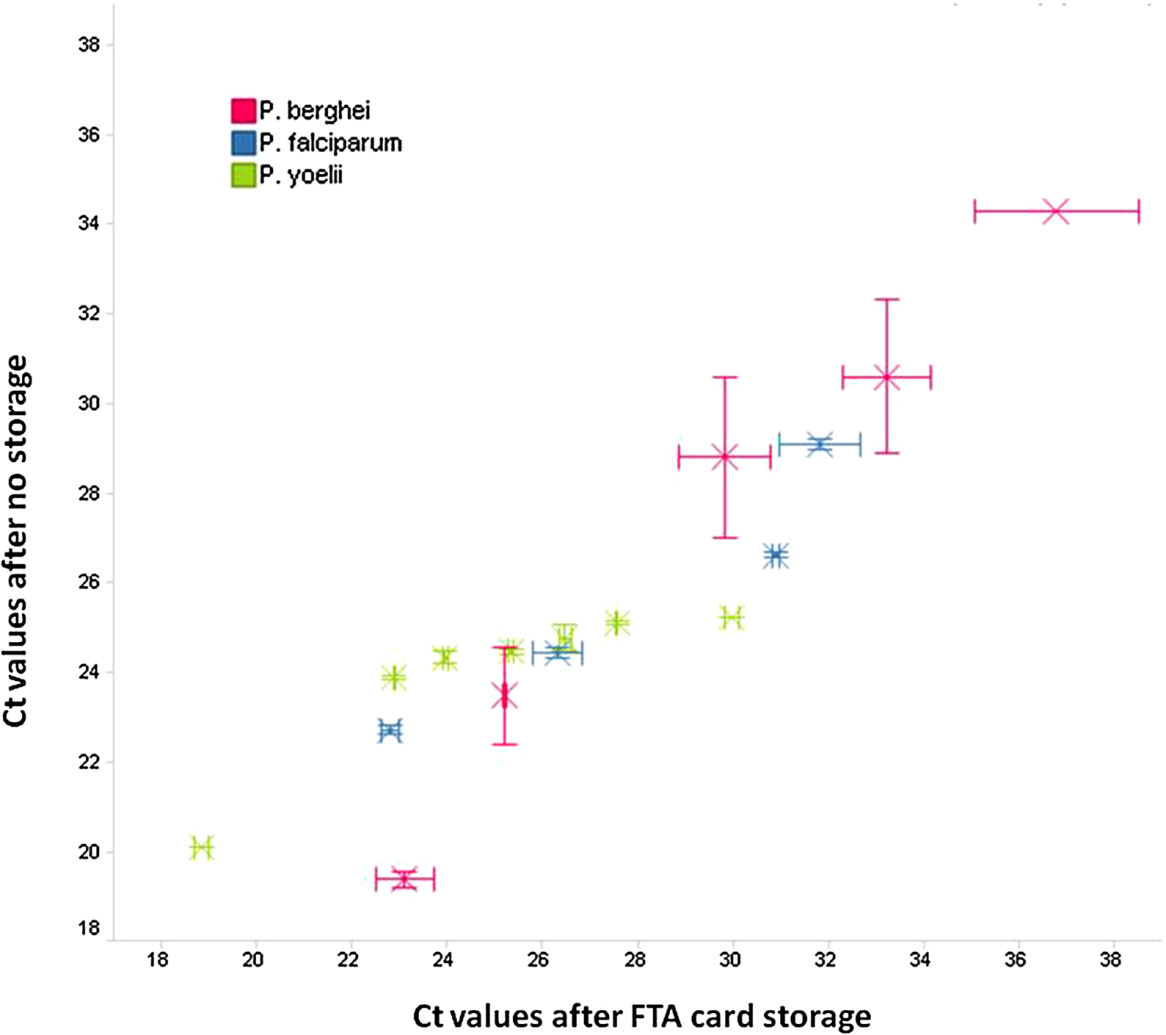
Plot of the Ct values obtained by qPCR with the CytB gene of samples processed directly after FTA embedding (no storage) or after storage for 52 months for *P. yoelii* and *P. falciparum* samples and for 26 months for *P. berghei*. *Error bars* correspond with the standard deviation of qPCR duplicates

The suitability of FTA cards for long-term storage of in vivo samples was addressed. FTA cards with *Plasmodium* samples were stored before DNA extraction at RT and protected from light and humidity for 52 months for *P. yoelii* and *P. falciparum* samples and for 26 months for *P. berghei*. The LOD (log10 parasites/µl) achieved in these cards using the CytB gene was 3.8 for *P. yoelii*, 3.2 for *P. falciparum,* and 1.3 for *P. berghei*. Figure 2 shows the correlation of the Ct values obtained from DNA extracted from FTA cards stored and from samples processed directly after FTA embedding without storage. *P. yoelii* and *P. falciparum* Ct values were similar at high parasitaemia levels or Ct under 24 and 26 respectively. For *P. berghei*, a linear correlation existed in all samples tested. The DNA yielded from the FTA cards stored met the quality requirements for qPCR, but rendered a lower LOD than the ones recently embedded.

## Discussion

Determination of parasitaemia by flow cytometry is accurate with a LOD of 0.0009 × 10^6^ parasites/µl but has the limitation of sample storage, and although microscopy is still considered the gold standard, it has a reduced throughput. Detection of *Plasmodium* DNA by amplification techniques based on PCR from dried blood on filter paper has been effectively used for sexual and asexual parasites even in studies conducted over decades [3, 9, 19, 20]. Studies performed with filter paper stored over long time periods and under different conditions have shown variable results as regards sensitivity for parasite detection [21]. Moreover, previous cross-platform work and cross-study comparisons already showed discrepancies on LOD in *P. falciparum* studies [22]. The study discussed here is the first to report use of filter paper with samples from mouse malaria models to support the early drug discovery process. The advantage of using FTA cards for in vivo assays has been shown, as they allow for sample storage at RT even for long time. In the present study, after extended storage periods at RT, although the LOD range was lower than for newly generated cards, the DNA extracted was sufficient for qualitative parasite detection, thus providing an advantage for retrospective studies.

The preanalytical steps of qPCR, and mainly DNA extraction, are extremely important because the quality of the genetic material collected impacts all downstream activities. Previous studies investigated different methods to optimize DNA extraction from filter papers [23–25]. In this study, and optimal DNA quality was obtained using the in-column silica membrane-based method. The qPCR technique has some limitations as routine methodology, including long sample processing, but was more sensitive than microscopy [10], powerful for detecting genes related to drug resistance, and amenable to parasite genotyping [2]. All these properties are critical in pre-clinical settings where access to and analysis of the original samples is crucial to identify potential drug failures.

Malaria-specific qPCR is being increasingly used because of its high sensitivity, speciation, and quantification of malaria parasites. In this study, although some qPCR efficiencies were slightly outside the desirable efficiency range of 90–105 %, quantification of *Plasmodium* parasites through qPCR with complete specificity has been shown even for samples with very low parasite densities using a set of primers for the most common analysed genes from murine *Plasmodium* species (*P. berghei* and *P. yoelii*) and the human pathogen studied in the humanized mouse model (*P. falciparum)*.

## Conclusions

This study has reported for the first time complete primer sets for parasite detection and quantification to be used in in vivo anti-malarial trials, and demonstrated the feasibility of using filter paper to collect and store in vivo animal samples.

The results of this study suggest that FTA cards are a practical and cost-effective, reliable source of *Plasmodium* DNA amenable to parasite detection and practical for studies of anti-malarial resistance genes and species identification. FTA cards require a low volume of sample and are easy to store over time. This technique represents a practicable tool for preclinical trials with anti-malarial drugs.
